# Hydrogen sulfide ameliorates senescence in vascular endothelial cells through ameliorating inflammation and activating PPARδ/SGLT2/STAT3 signaling pathway: Hydrogen sulfide ameliorates endothelial senescence

**DOI:** 10.3724/abbs.2023156

**Published:** 2023-08-16

**Authors:** Danyang Tian, Jinqi Meng, Lin Li, Hongmei Xue, Qi Geng, Yuxin Miao, Meng Xu, Ru Wang, Xiangjian Zhang, Yuming Wu

**Affiliations:** 1 Department of Physiology Hebei Medical University Shijiazhuang 050017 China; 2 Department of Sports Hebei Medical University Shijiazhuang 050017 China; 3 College of Pharmacy Hebei Medical University Shijiazhuang 050017 China; 4 Hebei Key Lab of Laboratory Animal Science Hebei Medical University Shijiazhuang 050017 China; 5 Hebei Collaborative Innovation Center for Cardio-Cerebrovascular Disease Shijiazhuang 050017 China; 6 The Key Laboratory of Neural and Vascular Biology Ministry of Education Shijiazhuang 050017 China; 7 Department of Pharmacology Tianjin Key Laboratory of Inflammatory Biology School of Basic Medical Sciences Tianjin Medical University Tianjin 300070 China

**Keywords:** hydrogen sulfide, endothelial cells, inflammation, senescence, peroxisome proliferator-activated receptor δ

## Abstract

Mounting evidence demonstrates that hydrogen sulfide (H _2_S) promotes anti-inflammatory molecules and inhibits pro-inflammatory cytokines in endothelial cells (ECs). This study aims to investigate the favorable action of H _2_S on endothelial function in senescence by inhibiting the production of inflammatory molecules. Senescent ECs exhibit a reduction in H _2_S, endothelial nitric oxide synthase (eNOS) and peroxisome proliferator-activated receptor δ (PPARδ), coupled with increased inflammatory molecules, sodium glucose transporter type 2 (SGLT2) and phosphorylation of STAT3, which could be reversed by the administration of a slow but sustained release agent of H _2_S, GYY4137. Decreased production of eNOS and upregulated p-STAT3 and SGLT2 levels in senescent ECs are reversed by replenishment of the SGLT2 inhibitor EMPA and the PPARδ agonist GW501516. The PPARδ antagonist GSK0660 attenuates eNOS expression and increases the production of p-STAT3 and SGLT2. However, supplementation with GYY4137 has no beneficial effect on GSK0660-treated ECs. GYY4137, GW501516 and EMPA preserve endothelial-dependent relaxation (EDR) in D-gal-treated aortae, while GSK0660 destroys aortic relaxation even with GYY4137 supplementation. In summary, senescent ECs manifest aggravated the expressions of the inflammatory molecules SGLT2 and p-STAT3 and decreased the productions of PPARδ, eNOS and CSE. H _2_S ameliorates endothelial dysfunction through the anti-inflammatory effect of the PPARδ/SGLT2/p-STAT3 signaling pathway in senescent ECs and may be a potential therapeutic target for anti-ageing treatment.

## Introduction

With the increasing number of ageing individuals and the extension of life expectancy in the general population worldwide, investigating how ageing contributes to progressively increasing sensitivity to morbidity, mortality, disability, and frailty has attracted substantial public attention [1]. The senescence of vessels plays an important role in the morbidity and mortality of the aging population and is relevant to diseases such as hypertension, atherosclerosis, and coronary heart disease [2]. The endothelium is a single layer of cells lining the blood vessels and maintains homeostasis of blood fluidity, vascular tone, and inflammatory immune responses. Senescent endothelial cells (ECs) usually undergo morphological alterations and induce endothelial dysfunction, contributing to cardiovascular diseases [3]. Pharmacological and non-pharmacological treatments are used to alleviate arterial ageing, but therapeutic approaches for cardiovascular diseases that are effective for young and middle-aged populations show reduced efficacy in older people. Therefore, effective treatments to improve the therapeutic effect of cardiovascular diseases, specifically in aged people, are urgently needed.

Older organisms contain high levels of pro-inflammatory molecules, which result in a chronic, systemic, and low-grade pro-inflammatory status. Interestingly, this phenomenon is referred to as ‘inflammaging’, which was first defined by Claudio Franceschi in 2000 [4]. Inflammaging in ECs disrupts vascular tone, augments endothelial permeability, impairs vascular integrity and mitochondrial biogenesis, and contributes to vascular stiffness and hypertension. Moreover, progressive endothelial senescence also promotes age-associated inflammation in a feedback manner [3].

Peroxisome proliferator-activated receptors (PPARs) belong to the nuclear hormone receptor family, which acts as transcription factors and transduces diverse cellular signals into cells, including environmental, inflammatory, and lipid and glucose metabolic signals. There are three PPAR isoforms in the body: PPARα, PPARβ/δ, and PPARγ, which play different regulatory roles [5]. PPARδ participates in the metabolism of lipids and cholesterol, modulation of glucose and fatty acid oxidation, and regulation of inflammation. Mounting evidence indicates that PPARδ preserves lipid metabolism and the progression of inflammation [6]. The administration of PPARδ agonists inhibits the upregulated expression of VCAM1 in ECs and IL1β, IL6 and TNFα in VSMCs by suppressing the phosphorylation of STAT3 [7]. These studies indicate that PPARδ plays an important anti-inflammatory role due to its transcriptional character. However, the role of PPARδ in the progression of inflammatory regulation in senescent ECs remains unclear.

SGLT2 inhibitors are a unique and novel class of anti-diabetic drugs that reduce glucose reabsorption in the renal tubule and promote urine excretion, ultimately decreasing plasma glucose levels [8]. Additional cardiovascular-preserving effects of SGLT2 inhibitors are detected when they are used in diabetic therapy. Clinical studies have proven that the SGLT2 inhibitors empagliflozin (EMPA), dapagliflozin (DAPA), and canagliflozin (CANA) present potential cardiovascular benefits in addition to their glucose-lowering effect [9]. DAPA induces weight loss and reduces systolic blood pressure by alleviating endothelial dysfunction and *ex vivo* vasorelaxation in pulmonary arteries. Chronic administration of DAPA preserves coronary artery vasorelaxation caused by SNP, which is an endothelium-independent effect, and ACh-mediated vasorelaxation, which is an endothelium-dependent effect in hyperglycemia [10]. Chronic supply of DAPA also enhances endothelial function in both adult and aged atherosclerotic animal models. CANA attenuates inflammatory signaling in HUVECs by activating AMPK and inhibiting IL1β [11]. Similar protective roles are observed in H _2_S-treated mice. H _2_S presents a plasma glucose lowering effect and regulates the metabolism of glucose in type 2 diabetes mellitus (T2DM) [12]. H _2_S preserves EDR in spontaneously hypertensive rats (SHRs) to lower blood pressure and downregulates autophagy of VSMCs via the AMPK/mTOR pathway to protect their function in T2DM [ 13, 14]. Various studies have demonstrated the cardiovascular protective role of SGLT2 inhibitors in addition to the hypoglycemic effect, which shows a similar effect of H _2_S.

H _2_S is a strong example of dose-dependent toxicity. The low intracellular concentration of H _2_S donates electrons to complex II of the mitochondrial electron transport chain, thereby stimulating ATP production. H _2_S acts as a gasotransmitter and plays an important role in the homeostatic mechanisms of the digestive, respiratory, urinary, nervous, and cardiovascular systems [15]. A huge number of the regulatory processes controlled by H _2_S are caused by persulfidation, which is an oxidative posttranslational modification of cysteine residues in proteins [16]. At high concentrations, H _2_S becomes toxic by binding to and inhibiting cytochrome C oxidase in complex IV of the electron transport chain to reduce the production of ATP and induce apoptosis [17]. In addition, a high concentration of H _2_S can serve as a substrate for complex II to provide electrons to mitochondria, which induces considerable oxidative stress [18]. In recent years, H _2_S has attracted great attention as a gasotransmitter because it regulates physiological processes in vascular cells, such as inflammation, oxidative stress, apoptosis, autophagy, the cell cycle, and mitochondrial metabolic functions [19]. Moreover, H _2_S promotes the production of anti-inflammatory molecules and inhibits the expression of pro-inflammatory cytokines in ECs and VSMCs. ECs respond to pathological conditions such as hypertension, hyperglycemia, hypercholesterolemia, and hyperhomocysteinaemia by promoting the production of ROS and inflammatory molecules that usually aggravate endothelial dysfunction and inhibit vascular relaxation [20]. H _2_S modifies endothelial function in SHRs, which is attributed to the suppression of inflammation through activating nuclear factor erythroid-2-related factor 2 [21]. Endogenous H _2_S deficiency causes inflammatory molecule overproduction in VSMCs and induces dilation dysfunction, leading to increased systolic pressure in mice [7].

Our previous study reported that decreased levels of H _2_S in aged mice are associated with increased oxidative stress and disrupt diurnal variation in cardiac function [22]. A previous review considered the impact of H _2_S on ageing, and the evidence showed that H _2_S is central to both physiological and pathological conditions, mediating protection across species and tissues. However, the relationship between H _2_S and inflammation in ECs in the aging process remains unclear. The present study aims to investigate the effect and possible mechanisms of the beneficial action of H _2_S on endothelial senescence during accelerated aging.

## Materials and Methods

### Animals

Male C57BL/6J mice aged 12 weeks were provided by Ex&InVivo Company (Shijiazhuang, China). All animals were kept in a room with a temperature of 20–24°C, humidity 60%±10%, and lighting with a 12-hour light/dark cycle. All mice were provided food and water ad libitum. All animal experiment procedures were performed following the Guide for the Care and Treatment of Laboratory Animals (NIH) and the Care and Use of Laboratory Animals approved by the Animal Care Committee of Hebei Medical University (Shijiazhuang, China).

### Aortic isolation and incubation

Animals were euthanized using carbon dioxide. All the operative instruments and body surfaces of animals were sterilized with 75% ethanol. Thoracic aortas were dissected from the surrounding adipose tissues, stored in sterile phosphate buffered solution (PBS), and cut into 1.6-mm length rings. Mouse aortic rings were incubated in an atmosphere of 5% CO _2_ at 37°C for 48 h in low-glucose DMEM (Gibco, Carlsbad, USA) containing 10% FBS (Gibco) and 1% antibiotics. Aortic rings were treated with D-gal (10‒100 mM; Sigma-Aldrich, St Louis, USA), GYY4137 (50‒400 μM; Cayman, Ann Arbor, USA), GW501516 (1 μM; MedChemExpress, Monmouth Junction, USA), GSK0660 (1 μM; MedChemExpress) or EMPA (0.5‒10 μM; MedChemExpress) for 48 h. Administration of 50 mM D-gal for 48 h was used to induce aortic senescence.

### Measurement of vascular relaxation

After incubation, the isometric force of the aortic rings was measured by the myograph in the Multi Myograph System (Danish Myo Technology A/S, Hinnerup, Denmark). Aortic rings were placed in Krebs solution at 37°C containing 119 mM NaCl, 4.7 mM KCl, 2.5 mM CaCl _2_, 1 mM MgCl _2_, 25 mM NaHCO _3_, 1.2 mM KH _2_ PO _4_, and 11 mM D-glucose in an environment with 95% O _2_ and 5% CO _2_. Before each experiment, each ring was stretched to 3 mN force and underwent 30 min of equilibration. The precontraction of the aorta was conducted using 60 mM K ^+^ and was removed by placing it in fresh Krebs solution. Then, a stable contraction by 1 μM phenylephrine (Phe) was obtained, and concentration-dependent relaxations evoked by accumulative acetylcholine (ACh; 0.003‒10 μM) were recorded and defined as EDR, representing endothelial function. Sodium nitroprusside (SNP; 0.001‒1 μM) caused relaxation after Phe contraction, which was determined as endothelium-independent relaxation.

### Cell culture and passage

The HUVEC line was obtained from Sciencell (Carlsbad, USA). HUVECs were cultured in endothelial cell medium (ECM, Sciencell) containing 5% FBS and 1% antibiotics in gelatin-coated flasks and maintained in an atmosphere of 5% CO _2_ at 37°C. HUVECs at passages 4‒6 with 80%‒90% confluence were used for the experiment. Six different batches of HUVECs were used in each group. Afterwards, administration of 50 mM D-gal (Sigma-Aldrich) for 48 h was used to induce HUVEC senescence.

### EdU proliferation assay

An EdU proliferation kit (RiboBio, Guangzhou, China) was used to determine cellular proliferation. HUVECs were plated in 96-well plates and treated with D-gal, GW501516, GSK0660, or EMPA for 48 h. Cells were incubated in ECM containing 50 μM EdU for 2 h. HUVECs were fixed in 4% paraformaldehyde (PFA) for 30 min, washed with 2 mg/mL glycine for 5 min and 0.5% Triton X-100 for 10 min. Then, the cells were stained with Apollo for 30 min and Hoechst 33342 for 30 min in the dark. Cycling cells were stained by Apollo, and the cell nuclei were stained by Hoechst 33342, and images were captured under a fluorescence microscope. Thereafter, the proliferative rate was calculated by the percentage of proliferating cells.

### Senescence-associated β-galactosidase staining

The senescence of HUVECs was determined by the SA-β-gal staining assay performed using the SA-β-gal staining kit (Beyotime Biotechnology, Shanghai, China). After being washed with PBS, the cells were fixed with 4% PFA. Then, the cells were stained with SA-β-gal staining solution at 37°C in an atmosphere without CO _2_ for 12 h and examined with a microscope (Leica, Heidelberg, Germany). The senescent cells were identified as blue-stained cells in the captured images.

### 2-NBDG uptake assay

Glucose uptake in the HUVECs was detected using 2-NBDG (Cayman). Cells were first deprived of glucose for 1 h in DMEM (Gibco) and then incubated in D-glucose-free DMEM with 100 μM 2-NBDG for 30 min. The cells were subsequently washed three times with PBS to cease 2-NBDG uptake and fixed with 4% PFA. The cell nucleus was labelled with DAPI. The 2-NBDG and DAPI fluorescence were measured with a fluorescence microplate reader (Molecular Devices, San Jose, USA).

### Western blot analysis

HUVECs were homogenized in RIPA lysis buffer and centrifuged at 20,000 *g* for 20 min at 4°C. Protein samples (10 μg) from independent experiments were electrophoresed on a 7.5%‒15% SDS-polyacrylamide gel and transferred onto PVDF membranes. Blots were blocked with 5% non-fat milk for 1 h at 37°C and incubated overnight at 4°C with antibodies against cystathionine gamma-lyase (CSE; sc-374249; Santa Cruz, Santa Cruz, USA), 3-mercaptopyruvate sulfotransferase (MPST; sc-374326; Santa Cruz), P21 (HA500005; HuaAn, Hangzhou, China), PPARδ (A5656; ABclonal, Shanghai, China), SGLT1 (A11976; ABclonal), SGLT2 (A20271; ABclonal), cystathionine beta-synthase (CBS; 14787-1-AP; Proteintech, Rosemont, USA), P53 (80077-1-RR; Proteintech), Caspase1 (WL03450; Wanlei, Shenyang, China), eNOS (WL01789; Wanlei), GLUT1 (WL01163; Wanlei), IL1β (WL00891; Wanlei), NLRP3 (WL02635; Wanlei), STAT3 (WL01836; Wanlei), and phosphorylated STAT3 (WLP2412; Wanlei). After being washed three times with TBST, the blots were incubated with anti-rabbit (SA00001-2; Proteintech) or anti-mouse IgG-HRP (SA00001-1; Proteintech). Antigen-antibody reactions were detected using ECL reagents (MERCK, Darmstadt, Germany) and detected with the ChemiScope 6100 system (CLiNX, Shanghai, China) for densitometric analysis.

### Statistical analysis

Data are expressed as the mean±SEM. Comparisons between two groups were performed using Student’s *t* test, whereas one-way ANOVA followed by the Bonferroni post hoc test was used when more than two groups were compared using Graphpad Prism (GraphPad Software, La Jolla, USA). *P*<0.05 was defined as statistically significant.

## Results

### D-gal induced endothelial senescence and inflammation and impaired endothelial function in HUVECs

**Figure 1:**
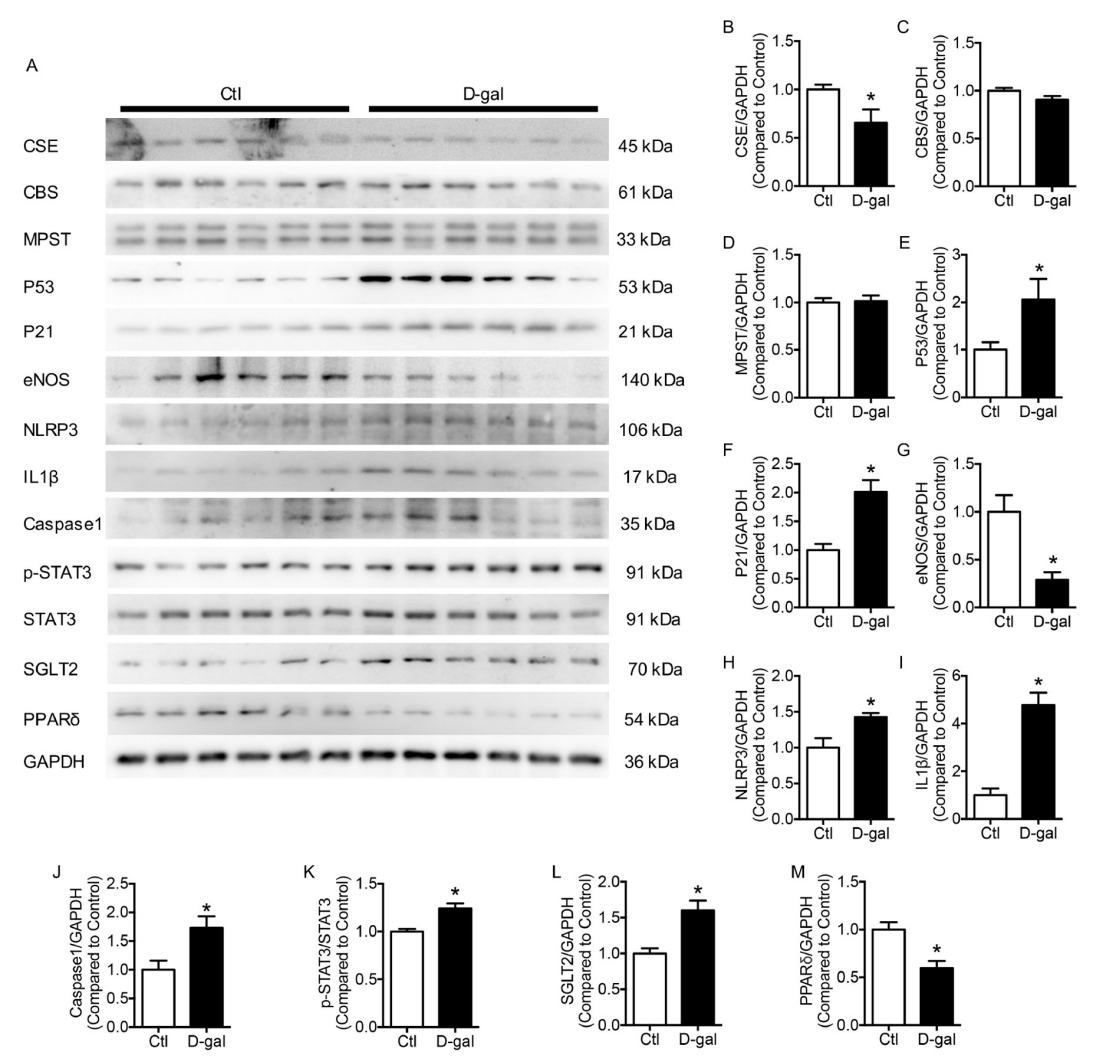
Figure 1 D-gal inhibits the production of H _2_S and induces cellular senescence and cellular inflammation in HUVECs (A–D) D-gal (50 mM, 48 h) inhibited the production of the H2S synthesis enzyme CSE but not CBS or MPST. (E,F) D-gal induced cellular senescence, demonstrating elevated P53 and P21 in HUVECs. (G) Expression of eNOS was decreased in the D-gal-treated group. (H–J) NLRP3, IL1β, and Caspase 1 were upregulated in D-gal-treated HUVECs. (K,L) The expression of phosphorylated STAT3 (Ser727) and SGLT2 was increased in the D-gal-treated group. (M) D-gal-treated HUVECs demonstrated decreased expression of PPARδ. Data are shown as the mean±SEM. n=6 in each group. Statistical differences were examined by unpaired Student’s t test. *P<0.05 vs control.

Treatment with D-gal for 48 h enhanced the percentage of SA-β-gal-positive cells, consistent with the overexpression of P53 and P21, and decreased the percentage of EdU-positive cells in a dose-dependent manner, which confirmed that *in vitro* stimulation of D-gal accelerated the senescence model at 50 mM D-gal exposure for 48 h ( Supplementary Figure S1A‒F). Compared with the control group, the H _2_S synthetic enzyme CSE, but not CBS or MPST, was diminished in D-gal-treated HUVECs ( Figure 1A–D). Elevated expression of the senescence markers P53 and P21 and impaired endothelial function demonstrated by the expression of eNOS were found in the D-gal group compared with the control group ( Figure 1E–G). To explore whether inflammatory molecules participates in the aging process, we performed western blot analysis and revealed that the expression levels of NLRP3, IL1β, and Caspase 1 were significantly elevated in the D-gal group ( Figure 1H–J). Phosphorylation of STAT3 and production of SGLT2 were upregulated in D-gal-treated HUVECs compared with those in the control HUVECs ( Figure 1K,L). However, decreased expression of PPARδ was observed in the D-gal group ( Figure 1M). These data suggested that D-gal-treated HUVECs exhibited an increased senescent phenotype and production of inflammatory molecules, impaired endothelial function, and decreased expression of PPARδ.

### H _2_S restored endothelial dysfunction and senescence in D-gal-treated aortas and HUVECs

**Figure 2:**
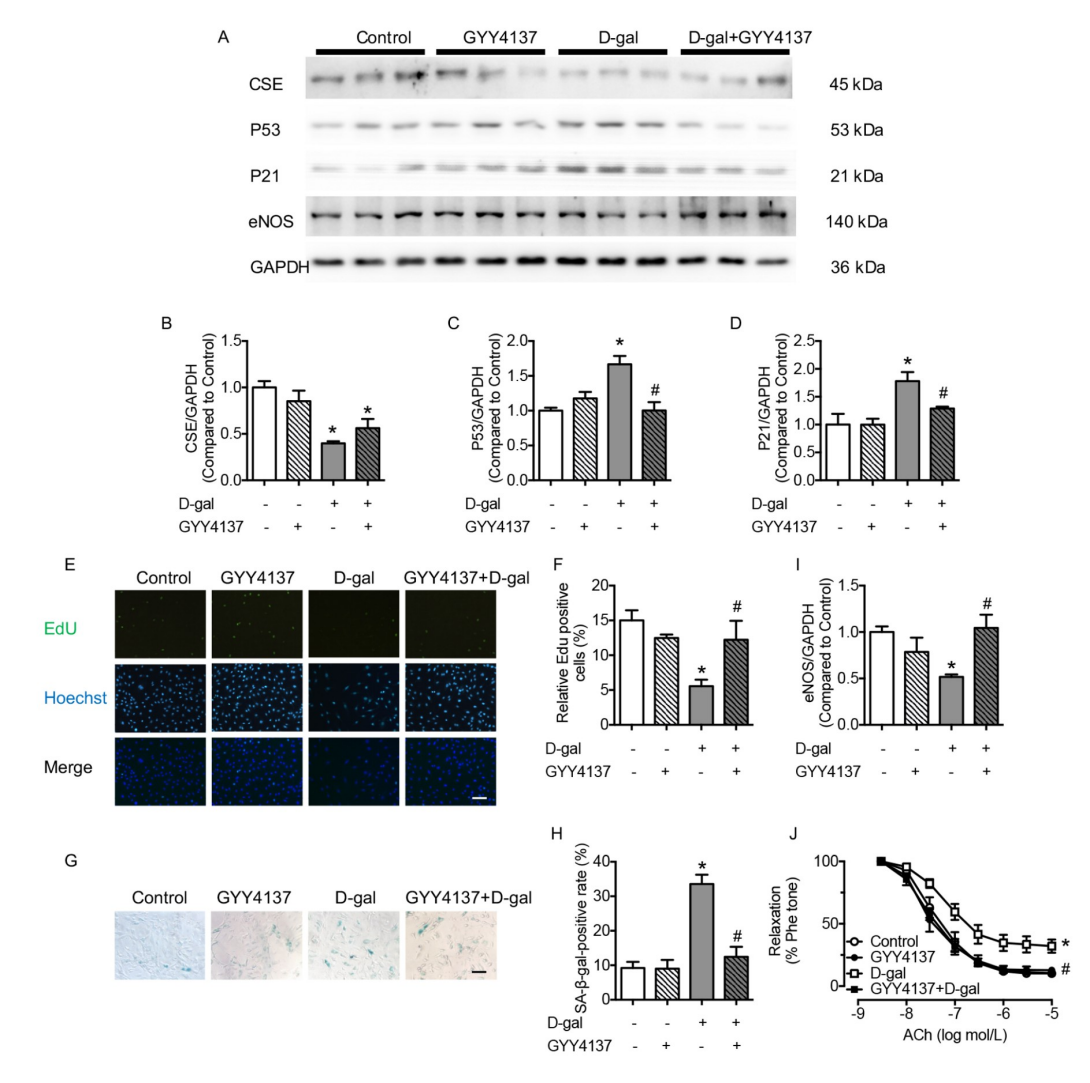
Figure 2 GYY4137 preserves endothelial function and cellular senescence (A) Representative western blots of CSE, P53, P21 and eNOS. (B) Decreased production of CSE in the D-gal group could not be improved by GYY4137 (200 μM, 48 h) supplementation. (C,D) Increased production of P53 and P21 in the D-gal group was reversed by GYY4137. (E,F) EdU (50 μM, 2 h), which is used to detect the proliferation of HUVECs, showed a decreased percentage of EdU-positive cells in the D-gal group and was elevated after GYY4137 supplementation (scale bar: 200 μm). (G,H) The percentage of SA-β-gal-positive cells was increased in D-gal-treated HUVECs and normalized by GYY4137 (scale bar: 200 μm). (I) Administration of GYY4137 normalized the decreased expression of eNOS in D-gal-treated HUVECs. (J) EDR was damaged by D-gal and was improved by GYY4137 treatment. Data are shown as the mean±SEM. n=6 in each group. Statistical differences were examined by one-way ANOVA with Tukey’s multiple comparisons test. *P<0.05 vs control, #P<0.05 vs D-gal.

**Figure 3:**
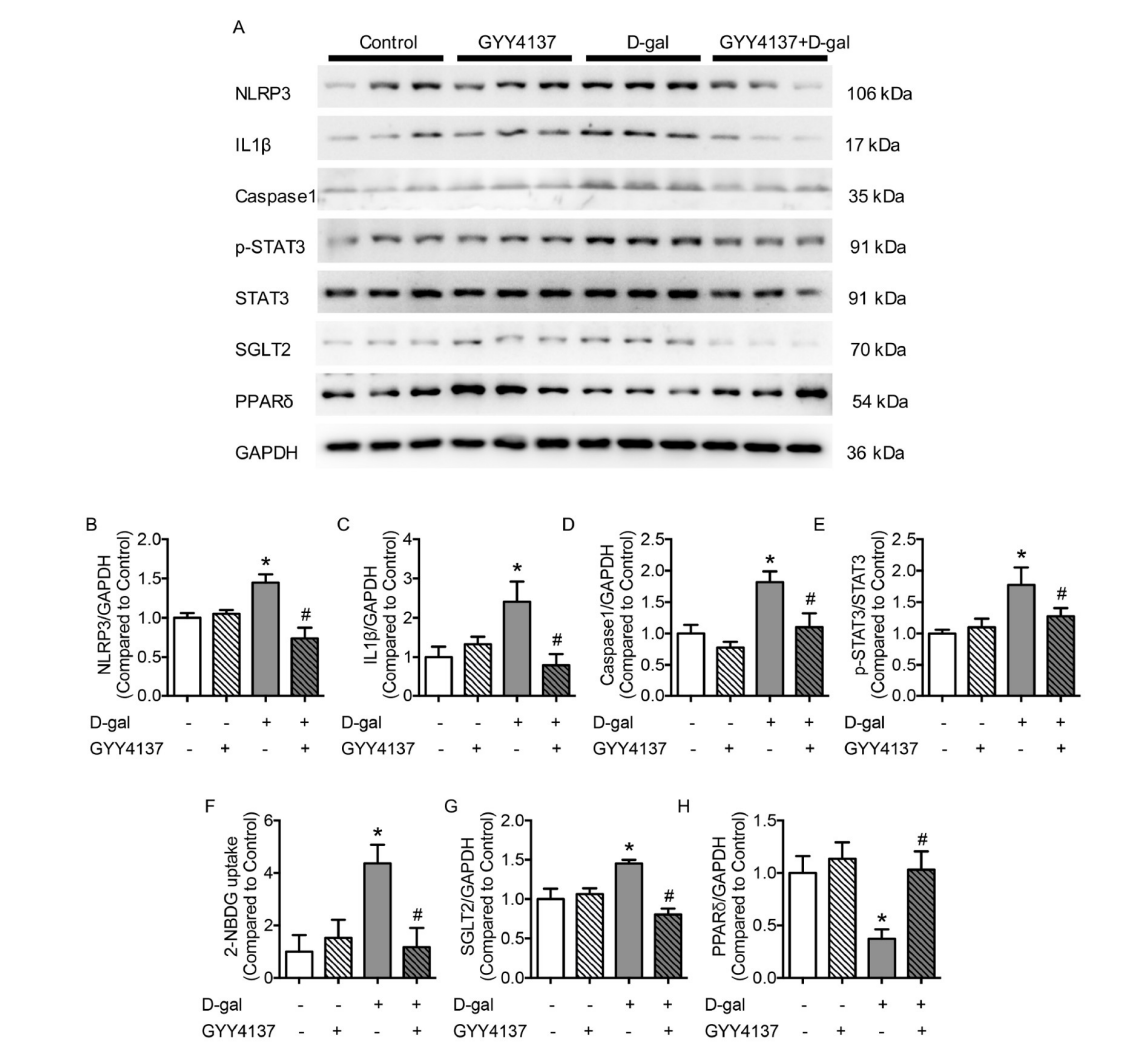
Figure 3 D-gal induces inflammation and phosphorylation of STAT3 and SGLT2 and diminishes PPARδ expression in HUVECs, which is corrected by GYY4137 (A) Representative western blots of NLRP3, IL1β, Caspase 1, p-STAT3, SGLT2, and PPARδ in HUVECs. (B–D) Expressions of the cellular inflammatory molecules NLRP3, IL1β, and Caspase 1, which are elevated in response to D-gal, could be reversed by co-incubation with GYY4137. (E) Increased production of p-STAT3 in the D-gal group is normalized by GYY4137. (F) HUVECs incubated with 2-NBDG (100 μM, 30 min) indicated that the increased 2-NBDG uptake in the D-gal group was normalized by GYY4137 treatment. (G) Increased SGLT2 production in the D-gal group is normalized by GYY4137. (H) GYY4137 improved the decreased expression of PPARδ in the D-gal group. Data are shown as the mean±SEM. n=6 in each group. Statistical differences were examined by one-way ANOVA with Tukey’s multiple comparisons test. *P<0.05 vs control, # P<0.05 vs D-gal.

D-gal induced cellular senescence and endothelial dysfunction. The compound GYY4137, which was used to provide a slow but sustained release of H _2_S, recovered the expressions of P53 and P21 in the D-gal group in a dose-dependent manner, confirming that 200 μM and 400 μM GYY4137 provided significant protection in preventing cellular senescence ( Supplementary Figure S2A,B). Then, HUVECs exposed to 200 μM GYY4137 for 48 h were used to determine the preserving effect of H _2_S on cellular senescence. Administration of GYY4137 did not affect the expression of CSE ( Figure 2A,B). H _2_S reversed the expressions of P53 and P21 and the percentage of SA-β-gal-positive cells, consistent with the restoration of EdU-positive cells in D-gal-treated HUVECs, demonstrating that H _2_S restored cellular senescence ( Figure 2C–H). Administration of GYY4137 improved the expression of eNOS in senescent HUVECs and the EDR in the D-gal-treated aorta ( Figure 2I,J), proving the endothelial protective effect of H _2_S in both aorta and HUVECs. No changes were observed in SNP-induced relaxation between different groups ( Supplementary Figure S4A). The expressions of the inflammatory molecules NLRP3, IL1β, Caspase 1, and the transcription factor p-STAT3 in the D-gal group were reversed by treatment with GYY4137, demonstrating the anti-inflammatory effect of GYY4137 ( Figure 3A–E). Exogenous administration of H _2_S restored endothelial inflammation in D-gal-treated HUVECs.

### H _2_S reversed the expressions of PPARδ and SGLT2 in D-gal-treated HUVECs

To determine the crucial protein that regulates the phosphorylation of STAT3, we detected the production of SGLT2 and PPARδ. The glucose uptake ability of HUVECs was determined by 2-NBDG, which was used as a fluorescent indicator representing augmented glucose uptake in the D-gal group ( Figure 3F). To evaluate which glucose transporter caused augmented glucose uptake in the D-gal-treated cells, the expression levels of GLUT1 and SGLT2, the principal glucose transporters in ECs, were measured. The production of SGLT2, which functions in glucose transportation, was augmented under D-gal treatment ( Figure 3G). However, no changes were observed in the case of GLUT1 (data not shown). The increased glucose uptake in senescent ECs was caused by overproduction of SGLT2, which was reversed by H _2_S. The expression of PPARδ, a crucial nuclear receptor regulating inflammation, was downregulated when treated with D-gal but recovered after GYY4137 treatment ( Figure 3H). Together, these results indicated that H _2_S restored cellular function in senescent ECs by preserving PPARδ production and inhibiting SGLT2 expression.

### PPARδ improved cellular senescence and preserved endothelial function in the aorta

**Figure 4:**
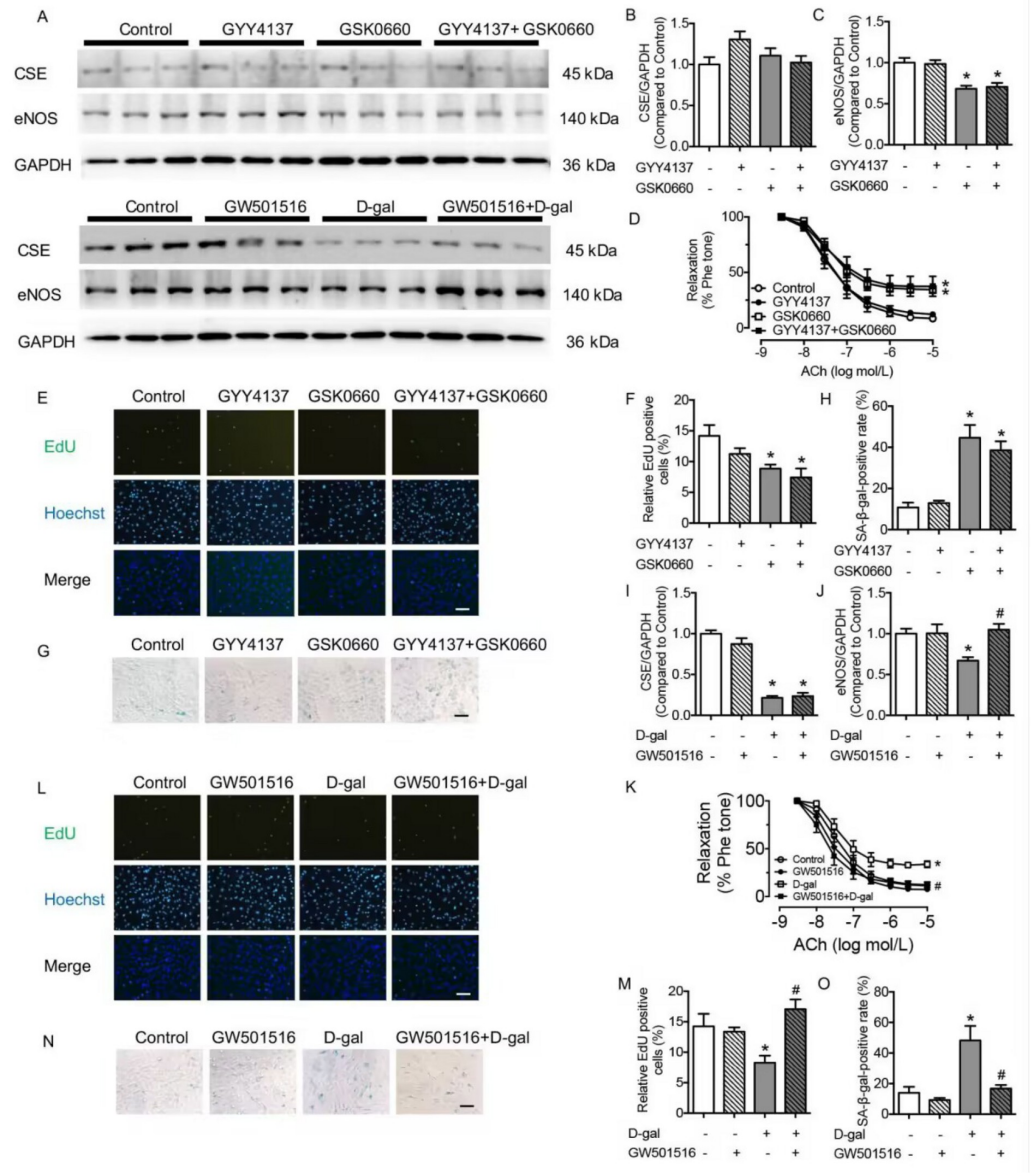
Figure 4 Inhibition of PPARδ abolishes the protective role of GYY4137 in HUVECs and impairs endothelial relaxation (A) Representative western blots of CSE and eNOS. (B) Administration of GYY4137 and GSK0660 (1 μM, 48 h) did not affect the expression of CSE. (C) GSK0660 diminished the expression of eNOS in HUVECs, and the effect could not be reversed by GYY4137. (D) EDR was damaged by GSK0660. (E,F) EdU, used to detect the proliferation of HUVECs, showed a decreased percentage of EdU-positive cells in the GSK0660 group, which did not change with GYY4137 exposure (scale bar: 200 μm). (G,H) The percentage of SA-β-gal-positive cells was increased in GSK0660-treated HUVECs, which was not altered by GYY4137 (scale bar=200 μm). (I) CSE expression was inhibited by D-gal and not affected by co-incubation with GW501516 (1 μM, 48 h). (J) Supplementation with GW501516 corrected the decreased expression of eNOS in D-gal-treated HUVECs. (K) Aortic EDR, damaged by D-gal, was reversed by GW501516. (L,M) The decreased percentage of EdU-positive cells in the D-gal group was elevated by GW501516 (scale bar: 200 μm). (N,O) The percentage of SA-β-gal-positive cells, decreased in D-gal-treated HUVECs, was normalized by GW501516 (scale bar: 200 μm). n=6 in each group. Statistical differences were examined by one-way ANOVA with Tukey’s multiple comparisons test. *P<0.05 vs control, #P<0.05 vs D-gal.

To determine whether PPARδ acts as a beneficial transcription factor in D-gal-induced cellular senescence, an agonist and an antagonist of PPARδ were applied to HUVECs and aortas. Neither GYY4137 nor GSK0660 affected the expression of CSE ( Figure 4A,B). GSK0660 abrogated the endothelial protection of GYY4137 by decreasing the production of eNOS in HUVECs and impairing the EDR of the aorta ( Figure 4C,D). However, SNP-induced relaxations showed no obvious changes between different groups ( Supplementary Figure S4B). A decreased percentage of EdU-positive staining and an increased percentage of SA-β-gal-positive cells were observed in PPARδ antagonist GSK0660-treated HUVECs, which could not be reversed by GYY4137 ( Figure 4E–H). Treatment with the antagonist of PPARδ accelerated cellular senescence and did not affect the expression of CSE. In contrast, the PPARδ agonist GW501516 did not affect CSE expression with or without D-gal treatment ( Figure 4I). The diminished expression of eNOS in HUVECs and damaged EDR of the aorta treated with D-gal were recovered by co-incubation with GW501516, demonstrating that PPARδ protected endothelial function in cellular senescence ( Figure 4J,K). The relaxations caused by SNPs were similar between different groups ( Supplementary Figure S4C). GW501516 increased the percentage of EdU-positive cells and decreased the percentage of SA-β-gal-positive cells in D-gal-treated HUVECs ( Figure 4L–O), suggesting that activating PPARδ improved D-gal-induced cellular senescence.

### PPARδ diminished the expression of SGLT2 and phosphorylation of STAT3 in HUVECs

**Figure 5:**
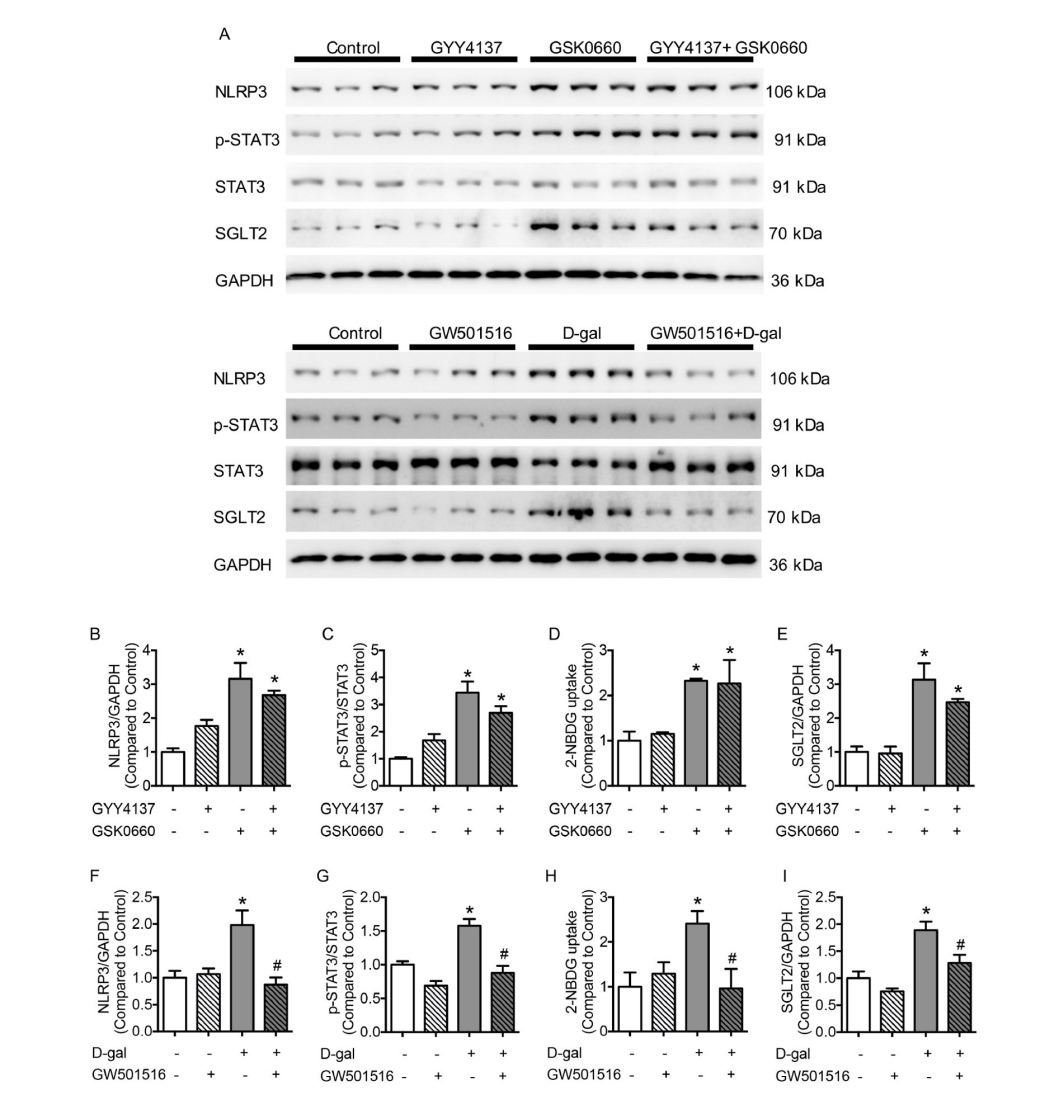
Figure 5 Activation of PPARδ preserves endothelial function and negates the expression of inflammatory molecules (A) Representative western blots of NLRP3, p-STAT3 and SGLT2. (B,C) The production of NLRP3 and p-STAT3 stimulated by GSK0660 was not affected by GYY4137. (D) Aggravated 2-NBDG uptake in GSK0660 cells cannot be normalized by GYY4137. (E) Expression of SGLT2 was increased in the GSK0660 group. (F,G) Increased production of NLRP3 and p-STAT3 in the D-gal group was reversed by GW501516. (H) Aggravated 2-NBDG uptake in the D-gal group was normalized by GW501516 treatment. (I) Increased production of SGLT2 in the D-gal group was reversed by GW501516. Data are shown as the mean±SEM. n=6 in each group. Statistical differences were examined by one-way ANOVA with Tukey’s multiple comparisons test. * P<0.05 vs control, #P<0.05 vs D-gal.

To determine the anti-inflammatory role of PPARδ and the regulatory effect of PPARδ on STAT3 in ECs, the expression of NLRP3 was detected when GW501516 or GSK0660 was used. When PPARδ was blocked by GSK0660, the expression of NLRP3 was significantly enhanced in HUVECs, and the changes could not be reversed by GYY4137 ( Figure 5A,B). Phosphorylation of the transcription factor STAT3 was elevated in the GSK0660 group compared with that in the control group, which was unaffected by GYY4137 ( Figure 5C). Glucose transportation was enhanced with the administration of GSK0660, as detected by the 2-NBDG uptake assay, and the trend of 2-NBDG changes was consistent with the expression changes of SGLT2 ( Figure 5D,E). The inhibition of PPARδ promoted the inflammatory response and the production of SGLT2. However, the overexpression of NLRP3 in the D-gal group was normalized by supplementation with GW501516. The single use of GW501516 did not affect the production of NLRP3 ( Figure 5F). Elevated phosphorylation of STAT3 in D-gal-treated HUVECs was normalized with GW501516 supplementation ( Figure 5G). When PPARδ was activated in the D-gal group with GW501516, the glucose uptake and the expression of SGLT2 were similar to the corresponding indexes of the control group ( Figure 5H,I). Therefore, our data indicated that PPARδ plays an anti-inflammatory role by inhibiting SGLT2 and phosphorylation of STAT3 in senescent cells and that the effect of PPARδ is independent of H _2_ S.

### The SGLT2 inhibitor EMPA reversed cellular senescence and preserved endothelial function in the aorta

**Figure 6:**
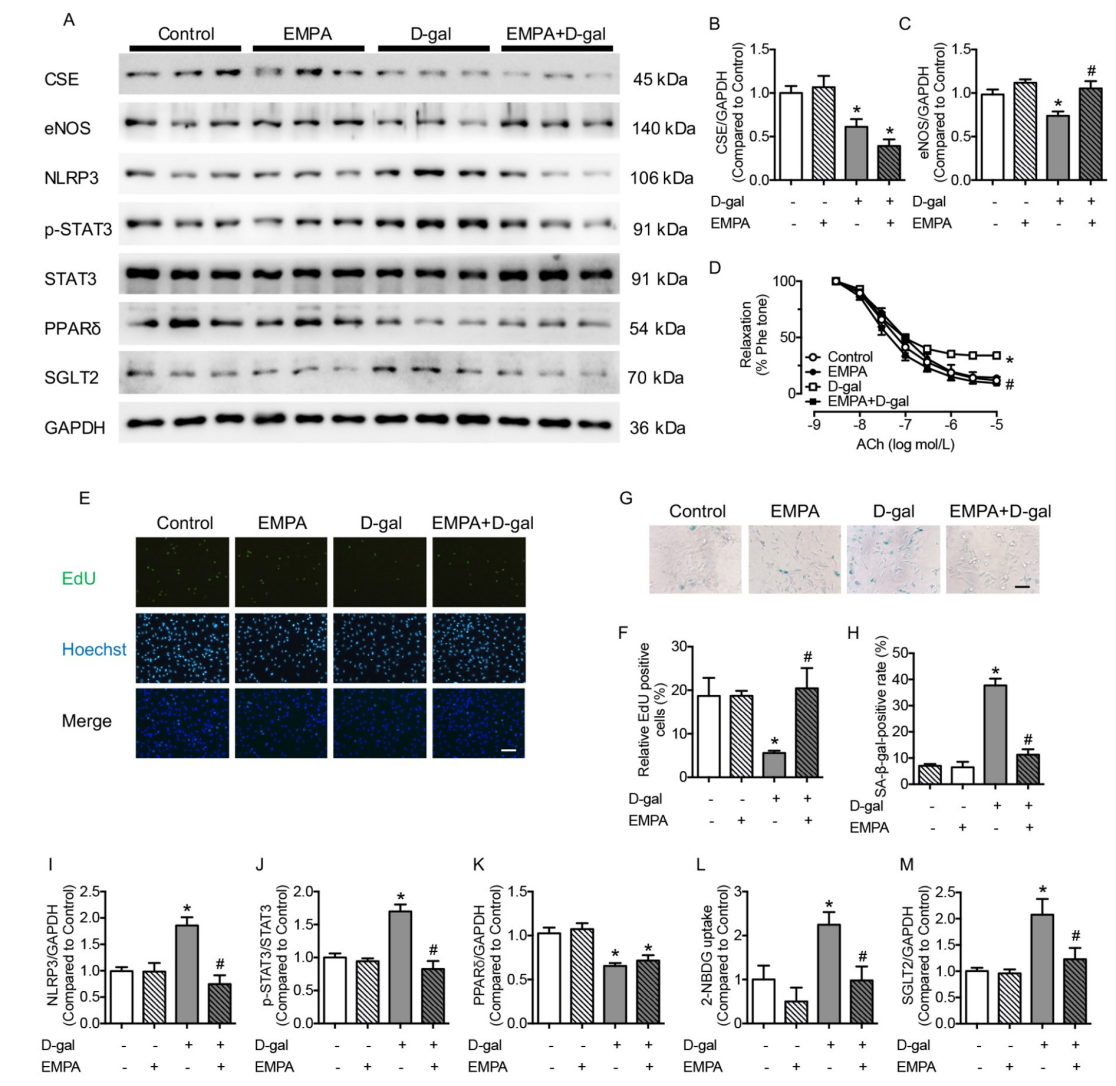
Figure 6 SGLT2 inhibitor EMPA preserves cellular senescence, negates inflammatory molecule expressions in D-gal treated HUVECs, and corrects impaired EDR in the D-gal treated aorta (A) Representative western blots of CSE, eNOS, NLRP3, p-STAT3, PPARδ and SGLT2. (B) CSE expression inhibited by D-gal was reversed by co-incubation with EMPA (5 μM, 48 h). (C) Supplementation with EMPA corrected the decreased expression of eNOS in D-gal-treated HUVECs. (D) EDR of the aorta, damaged by D-gal, was reversed by EMPA. (E,F) The decreased percentage of EdU-positive cells in the D-gal group was increased by EMPA (scale bar: 200 μm). (G,H) The percentage of SA-β-gal-positive cells, decreased in D-gal-treated HUVECs, was normalized by EMPA (scale bar: 200 μm). (I,J) Increased production of NLRP3 and p-STAT3 in the D-gal group was reversed by EMPA treatment. (K) Supplementation with EMPA reversed the decreased expression of PPARδ in D-gal-treated HUVECs. (L) Aggravated 2-NBDG uptake in D-gal was normalized by EMPA treatment. (M) Increased production of SGLT2 in the D-gal group was reversed by EMPA treatment. Data are shown as the mean±SEM. n=6 in each group. Statistical differences were examined by one-way ANOVA with Tukey’s multiple comparisons test. *P<0.05 vs control, #P<0.05 vs D-gal.

To investigate the regulatory mechanism of PPARδ, SGLT2, and p-STAT3, the SGLT2 inhibitor EMPA was used, and the cellular senescence and endothelial function of the aorta were detected. EMPA (5 μM and 10 μM) substantially inhibited SGLT2 production in the D-gal-treated group, which was in accordance with the expressions of P53 and P21. However, the changes in SGLT2 production presented the opposite tendency with the expression of eNOS in the D-gal and EMPA groups ( Supplementary Figure S3A‒D). Administration of 5 μM EMPA did not change CSE expression with or without D-gal stimulation ( Figure 6A,B). The decreased expression of eNOS and damaged EDR of the aorta in the D-gal group were improved by EMPA co-incubation, demonstrating that the damaged endothelial function in senescence was preserved by inhibiting SGLT2 ( Figure 6C,D). There were no changes in SNP-induced relaxations in different groups ( Supplementary Figure S4D). The decreased percentage of EdU-positive staining and enhanced percentage of SA-β-gal-positive cells in D-gal-treated HUVECs were normalized by the administration of EMPA, demonstrating that the inhibition of SGLT2 preserved the cellular senescence caused by D-gal ( Figure 6E–H). The results indicated that inhibition of SGLT2 modified endothelial function and cellular senescence without affecting the expression of CSE.

### EMPA inhibited the expression of inflammatory molecules and phosphorylation of STAT3 in HUVECs

As an SGLT2 inhibitor, EMPA inhibited the production of the inflammatory molecule NLRP3 in D-gal-treated HUVECs ( Figure 6I), and the phosphorylation of STAT3 was also attenuated by EMPA treatment ( Figure 6J). Therefore, inhibition of SGLT2 reduced the expression of inflammatory molecules and p-STAT3. To determine the regulatory pathway of PPARδ and SGLT2, the expression levels of PPARδ and SGLT2 were detected in the presence of EMPA. EMPA could not regulate the production of PPARδ in D-gal-treated HUVECs ( Figure 6K). However, EMPA inhibited the increased SGLT2 production in the D-gal group and attenuated the enhanced glucose transportation in senescent cells ( Figure 6L,M). These results indicated that SGLT2 inhibition diminished inflammation, phosphorylation of STAT3, expression of SGLT2, and glucose uptake. PPARδ inhibited STAT3 phosphorylation by decreasing the production of SGLT2 in senescent cells.

## Discussion

Cellular senescence is an essential aging process in response to various endogenous and exogenous stressors, such as ROS, DNA damage, and inflammation, resulting in phenotypic alterations and functional dysfunction. Endothelial senescence is involved in the pathogenesis of heart failure, diabetes, atherosclerosis, and age-related diseases [ 23, 24]. In addition to regulating vascular resistance and tissue perfusion, endothelium-derived nitric oxide (NO) also exerts pivotal vaso- and cardio-protective effects [25].

The expression of eNOS is decreased in senescent HUVECs. Previous studies demonstrated that the reduced bioavailability of NO is involved in the age-related reduction in EDR. We examined EDR and observed that ACh-induced relaxation was impaired in the D-gal-treated aorta, which could be normalized by GYY4137, an H _2_S supplement to provide a sustained release of H _2_S for 24 h. However, the SNP-induced relaxations showed no difference between different groups, proving that impaired relaxation of senescent aorta is caused by endothelium but not VSMCs. Mounting evidence shows that endothelial dysfunction generated by increased inflammation distinctly contributes to injured vascular dilation and is a risk factor for cardiovascular diseases [26]. The level of inflammatory molecules is elevated in senescent cells, which increases cellular senescence [27]. The NLRP3 inflammasome plays a pivotal role in vascular inflammation. Activated NLRP3 undergoes oligomerization and activates Caspase 1, serving as an enhancer of multiple pro-inflammatory pathways, including NF-κB, COX-2/PGE2, and ROS [28]. Our results demonstrated that the expression of NLRP3 was increased in D-gal-treated HUVECs accompanied by Caspase 1 and IL1β, and the expression of NLRP3 could be reversed by co-incubation with GYY4137, suggesting that H _2_S could improve endothelial function by inhibiting endothelial inflammation.

H _2_S is an endogenous biological gasotransmitter molecule produced by three enzymes: CSE, CBS, and MPST, which catalyze L-cysteine to generate H _2_S [29]. CBS is mainly expressed in the central nervous system [30], MPST is mainly localized in smooth muscle cells and cardiomyocytes [31], and CSE is produced mainly in blood vessels and the heart; therefore, the changes in CSE in ECs present the variation tendency of H _2_S. Decreased expression of CSE and no obvious changes in CBS or MPST were found in D-gal-treated HUVECs. The level of H _2_S is decreased in senescent HUVECs, proving that the decreased production of H _2_S in senescent ECs is coordinated with the changes in CSE expression. In some studies, GYY4137 was reported to induce H _2_S release from endothelial cells. However, preincubation of the arteries with an inhibitor of CSE, PPG, did not change the dose-response curve of GYY4137. In addition, in the presence of arterial tissue, the amount of sulfides measured from GYY4137 was not increased, suggesting that H _2_S release is tissue-independent [32]. We also noticed that the expression of CSE was not affected by the administration of GYY4137, which is consistent with previous studies, demonstrating that GYY4137 is only used as a donor to give a slow release of H _2_S.

Administration of GYY4137 suppressed glucose transportation in D-gal-treated HUVECs. Glucose transportation examined by the 2-NBDG assay showed increased glucose transportation in senescent HUVECs, and we wondered which kind of transporter mediates glucose transportation [33]. There are two classes of carrier proteins that reabsorb glucose: SGLTs and glucose transporters (GLUTs). SGLTs, which are located on the luminal surface of the proximal tubule epithelium, actively transport glucose into cells against a concentration gradient driven by the cotransportation of sodium and ATP consumption. However, GLUT transporters located on the proximal tubule transport glucose passively following the concentration gradient [34]. We detected the expression of GLUT1, which is the major type of glucose transporter expressed in HUVECs, and no significant differences in GLUT1 between young and senescent cells were observed [35]. SGLTs include SGLT1 and SGLT2, and the expression of SGLT1 was not detected in HUVECs, demonstrating that the major glucose transporter in ECs is SGLT2, which is consistent with a previous study [36]. The increased 2-NBDG uptake in senescent cells was inhibited by the SGLT2 inhibitor EMPA, which also confirmed that glucose uptake is conducted by SGLT2 in HUVECs but not SGLT1 or GLUT1. H _2_S preserved the endothelial function of senescent cells by inhibiting the expression of SGLT2 and suppressing glucose transportation. Many clinical studies revealed an age-associated increase in glucose uptake but a decrease in glucose utilization, which is consistent with our results [37]. In aging cells, there is a switch of energy metabolism from aerobic oxidative metabolism to anaerobic glycolysis, which leads to much less production of ATP. Therefore, the decrease in energy production in senescent cells, which is inadequate for the cells, causes a compensatory uptake of glucose, manifesting as an obvious increase in 2-NBDG uptake in senescent endothelium.

Impaired EDR in the D-gal-treated aorta, enhanced glucose transportation and elevated endothelial-inflammatory molecules in senescent HUVECs were normalized by administration with EMPA. Glucose is a critical component in the pro-inflammatory response, and research with macrophages verified that elevated glucose uptake and metabolism modulated the inflammatory response and that this effect could be blunted by pharmacologic inhibition of glycolysis [38]. Initially, we regarded this endothelial impairment in the senescent aorta and ECs as the increased level of blood glucose, and EMPA preserved the EDR in senescent aorta by lowering the level of blood glucose. However, the plasma glucose concentration presented no obvious differences between young and senescent groups, indicating that the improvement of endothelial function in EMPA-treated aging ECs is independent of lowering blood glucose [39]. DAPA attenuated myocardial ischemia/reperfusion injury by limiting NLRP3 inflammasome activation and modulating autophagy independently of its hypoglycemic effect [40]. Elevated production of NLRP3 and phosphorylation of STAT3 in senescent cells were normalized by EMPA supplementation. EMPA reduced myocardial infarction and lipid peroxidation in a STAT3-dependent manner. STAT3 is a crucial promoter of inflammatory molecules regulating the expressions of TNFα, IL6, and IL1β [7]. The NLRP3 inflammasome and IL1β production can be blocked by interfering with the JAK/STAT signaling pathway [41]. These results illustrated that elevated SGLT2 damages endothelial function through the STAT3/NLRP3 inflammatory response in senescent cells, which is reversed by supplying H _2_S and inhibiting the SGLT2/STAT3/NLRP3 signaling pathway.

PPARδ is a nuclear receptor that suppresses the inflammatory reaction. PPARδ activation in perirenal fat by agonists inhibits SGLT2 function, which is mediated by increased production of adipose adiponectin [42]. In our study, the agonist of PPARδ, GW501516, decreased the expression of SGLT2 and glucose transportation in senescent ECs and preserved the relaxation of D-gal-treated aortas. GSK0660, an antagonist of PPARδ, upregulated the expression of SGLT2 and glucose transportation in ECs and impaired the relaxation of the aorta. The administration of GYY4137 did not change the elevated protein production and the impaired endothelial function caused by GSK0660. The SGLT2 inhibitor EMPA did not affect the expression of PPARδ, indicating that the transcription factor PPARδ regulates SGLT2 transcription. PPARδ is a nuclear receptor that suppresses the inflammatory reaction, which is in accordance with the anti-inflammatory role of H _2_S [7]. H _2_S upregulated the expression of PPARδ in the renal arterial endothelium in hypertensive rats [13]. The production of PPARδ was inhibited in senescent cells, which could be reversed by GYY4137, demonstrating that H _2_S regulated the production of PPARδ. However, the expression of CSE was not affected by GW501516, GSK0660, or EMPA. The inhibitory effect of H _2_S on the expression of SGLT2 in senescent cells depended on PPARδ. These results illustrated that the decreased PPARδ promoted the expression of SGLT2 in senescent cells, which was reversed by H _2_S through activating the PPARδ-mediated SGLT2/STAT3/NLRP3 signaling pathway.

The novelty of the present study is that it first demonstrates that H _2_S inhibits SGLT2 expression in senescent HUVECs and improves EDR in senescent aortas. H _2_S inhibits the expression of SGLT2 in a PPARδ-dependent manner and thus inhibits the phosphorylation of STAT3. SGLT2 inhibitors are usually used as diabetic therapy drugs, and cardiovascular protection has been reported clinically. In the present study, the SGLT2 inhibitor EMPA also preserved endothelial function by inhibiting inflammatory molecules. All these findings prove that H _2_S improves endothelial function by suppressing the expression of SGLT2 in senescent ECs. This study provides new insights regarding the favorable role of H _2_S in senescent ECs and indicates that H _2_S might be a new therapeutic target for ageing. However, the mechanisms of how PPARδ regulates SGLT2/p-STAT3 have not been investigated clearly, and further studies are required to prove the beneficial effect of PPARδ on aging.

## Supporting information

651Supplementary_Figures

## Supplementary Data

Supplementary data is available at *Acta Biochimica et Biophysica Sinica* online.
