# Supplementary material for: Hydrogen sulfide ameliorates senescence in vascular endothelial cells through ameliorating inflammation and activating PPARδ/SGLT2/STAT3 signaling pathway: Hydrogen sulfide ameliorates endothelial senescence
Source: Acta Biochim Biophys Sin (Shanghai). 2023 Aug 16;55(9):1358–69. doi: 10.3724/abbs.2023156 (PMC10520484; doi:10.3724/abbs.2023156)
Supplement: 651Supplementary_Figures [file 651Supplementary_Figures.pdf]

Supplementary Figures

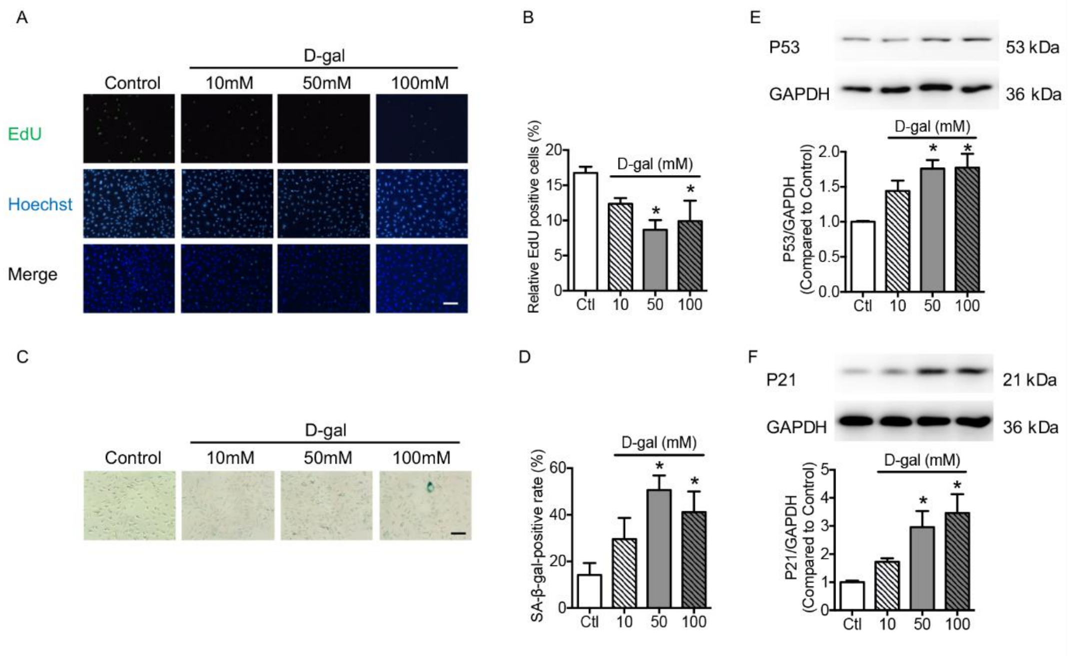

Supplementary Figure S1. D-gal induces cellular senescence in a dose-dependent manner (A, B) D-gal decreased the percentage of EdU (50 μM, 2 h)-positive cells in a dose-dependent manner. (C, D) D-gal increased the percentage of SA-β-gal-positive cells in a dose-dependent manner. (E, F) The expression of P53 and P21 was increased with D-gal treatment in a dose-dependent manner. Data are shown as the mean ± SEM. *n*=6 in each group. Statistical differences were examined by one-way ANOVA with Tukey's multiple comparisons test. $^{*}P<0.05$ vs control.

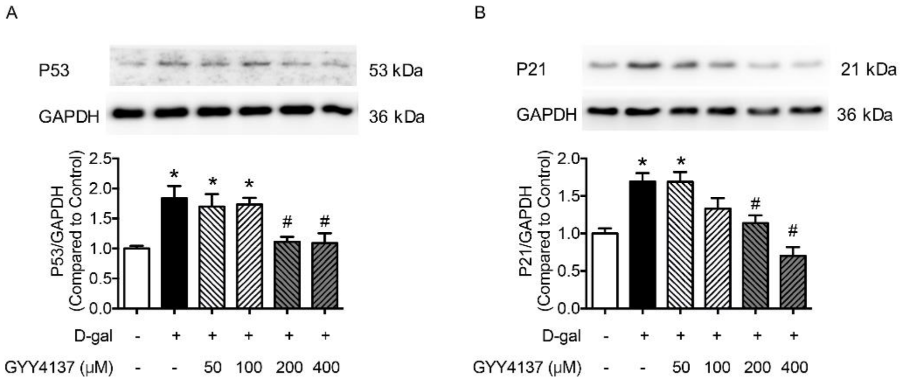

Supplementary Figure S2. GYY4137 improves cellular senescence in a dose-dependent manner (A, B) Increased expression of P53 and P21 in D-gal cells wa normalized by GYY4137 in a dose-dependent manner (50-400 μM, 48 h). Data are shown as the mean ± SEM. n=6 in each group. Statistical differences were examined by one-way ANOVA with Tukey's multiple comparisons test. $^{*}P$<0.05 vs control, $^{\#}P$<0.05 vs D-gal.

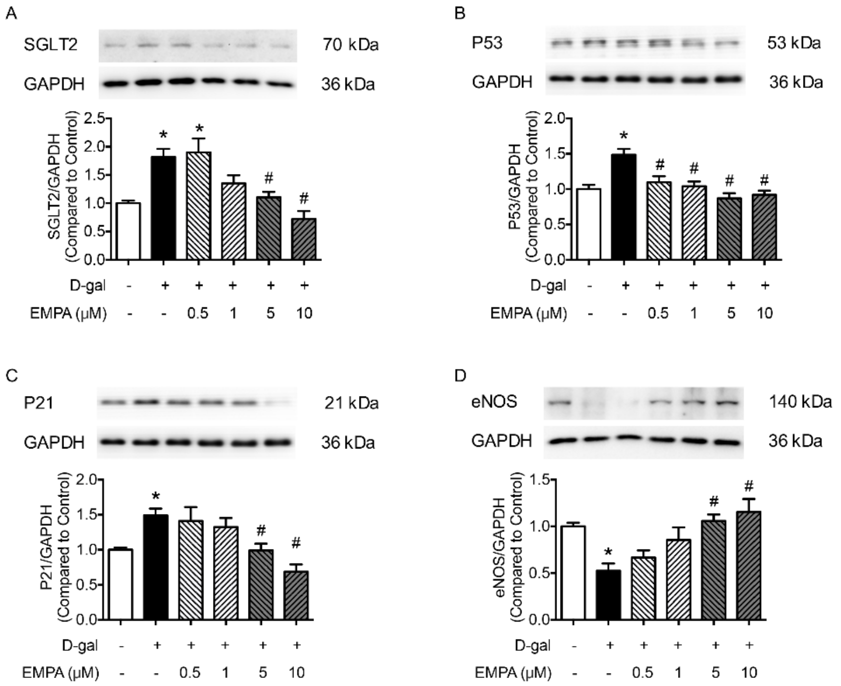

Supplementary Figure S3. EMPA improves cellular senescence and inflammation and preserves endothelial function (A) EMPA inhibited SGLT2 expression in D-gal-treated HUVECs in a dose-dependent manner (0.5-10 μM, 48 h). (B, C) EMPA improved D-gal-induced cellular senescence in a dose-dependent manner. (D) EMPA preserved endothelial function by upregulating the expression of eNOS in the D-gal group in a dose-dependent manner. Data are shown as the mean ± SEM. *n*=6 in each group. Statistical differences were examined by one-way ANOVA with Tukey's multiple comparisons test. $^{*}P<0.05$ vs control, $^{\#}P<0.05$ vs D-gal.

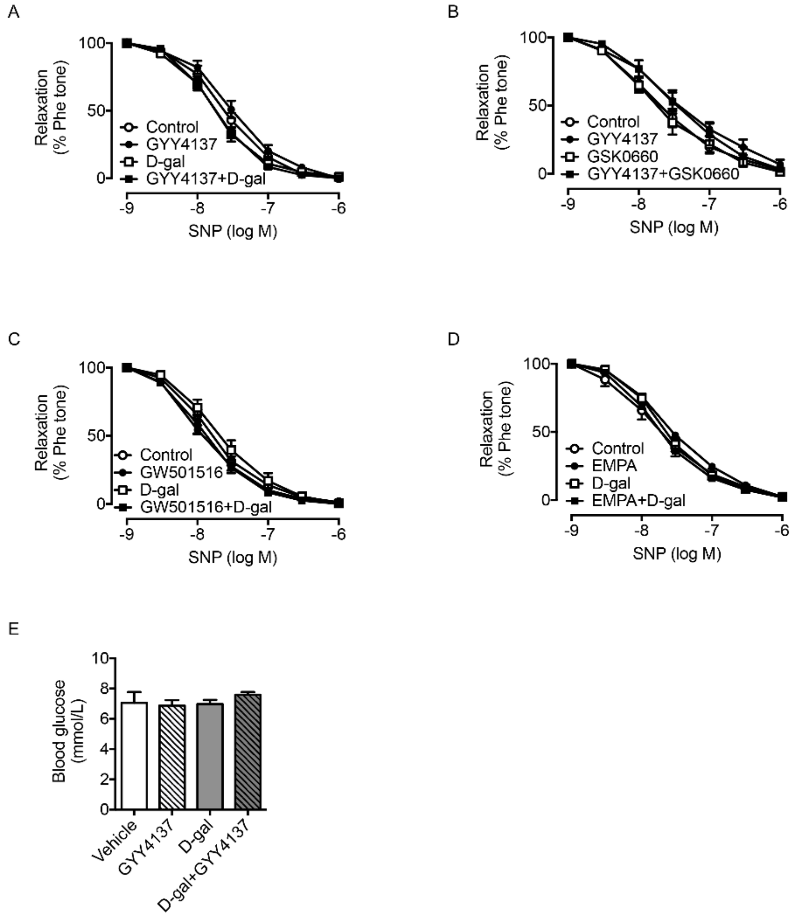

Supplementary Figure S4. Endothelium-independent relaxation and blood glucose represent no difference between groups (A–D) Administrations of GYY4137, D-gal, GSK0660, GW501516 or EMPA did not change the SNP-evoked relaxation compared with the control. (E) Blood glucose of GYY4137- and D-gal-treated mice. Data are shown as the mean ± SEM. *n*=6 in each group. Statistical differences were examined by one-way ANOVA with Tukey's multiple comparisons test. $^{*}P<0.05$ vs control.
